# Treatment of Cholera

**Published:** 1849

**Authors:** 


### Article V.—*Treatment of Cholera.*

Dr. Maxwell of Calcutta, who has lately published a "Key to Cholera," (he himself having had three attacks of the disease,) thus alludes to his recovery from the last attack. Our quotation commences after his description of the occurrence of the characteristic spasms.

"The thirst, however, became worse and worse, and I determined to relieve it at all hazards, and not add misery to death. Having made up my mind, the next point was to choose the particular beverage; there was plain water, whey and barley-water, gruel, congee, &c., wine and water, brandy and water, &c. To the last of these I had a repugnance, as every one has in full formed Cholera, and the others would require time and direction for their preparation, which my disease was not able to afford, or I to give. Whilst thus ruminating, my eye accidentally fell upon a packet of effervescing soda powders standing among a crowd of other remedies and nostrums on the table. It immediately struck my fancy; it struck me as the very thing I wanted, and without further delay, I pointed to it, and made signs for a copious draught thereof. It was soon made, and soon swallowed; it was extremely refreshing and agreeable, and the thirst was allayed; no nausea succeeded, and the pleasing anticipation remained of having a repetition of the draught whenever I desired. This I was not long in desiring; in fact, almost immediately afterwards I swallowed another, and continued repeating it whenever the thirst became urgent. Instead of retrograding or remaining stationary, I began to improve; the stools became easier, and the spasms less vigorous and vicious.

I experienced an inclination to sleep, a desire to be covered up, and for something hot to drink—(these are the best signs, as they point out the disease escaping from the collapse stage). I had a large tumblerfull of warm but very weak brandy and water made, and drank it off. I fell asleep, and had five or six hours sound repose. I awoke bathed in perspiration, and with the exception of a little stiffness and considerable thirst, I felt perfectly well. The thirst was again relieved by the effervescing draughts and I followed up the principle with a couple of dishes of that most delectable and pre-eminent of all stomachics, tea."—*Medical Times* in *N. J. Med. Rep.*

